# The effect of anesthetic technique on µ-opioid receptor expression and immune cell infiltration in breast cancer

**DOI:** 10.1007/s00540-018-2554-0

**Published:** 2018-09-18

**Authors:** Kirk J. Levins, S. Prendeville, S. Conlon, D. J. Buggy

**Affiliations:** 1 Department Anesthesia, Mater Misericordiae University Hospital, Dublin, Ireland; 2 Department Pathology, Mater Misericordiae University Hospital, Dublin, Ireland; 3 School of Medicine, University College Dublin, Dublin, Ireland; 4 Department of Outcomes Research, Cleveland Clinic, Cleveland, OH USA

**Keywords:** Cancer, Anesthesia, Regional anesthesia, Breast cancer metastasis, µ-Opioid receptor in cancer

## Abstract

**Background:**

Clinical histological studies demonstrate that the distribution of natural killer (NK) cells, other immune cells and μ-opioid receptors (MOR) within cancer tissue can predict cancer prognosis. No clinical study has evaluated whether anesthetic technique influences immune cell and MOR expression within human breast cancer.

**Methods:**

Excised preoperative biopsies and intraoperative breast cancer specimens from 20 patients randomly chosen from patients previously enrolled in an ongoing, prospective, randomized trial (NCT00418457) investigating the effect of anesthetic technique on long-term breast cancer outcome were immunohistochemically stained and microscopically examined by two independent investigators, masked to randomization, to quantify MOR and immune cell infiltration: CD56, CD57 (NK cells), CD4 (T helper cells), CD8 (cytotoxic T cells) and CD68 (macrophages). Patients had been randomized to receive either a propofol–paravertebral anesthetic with continuing analgesia (PPA, *n* = 10) or balanced general anesthetic with opioid analgesia (GA, *n* = 10).

**Results:**

There were no differences between the groups in staining intensity in preoperative biopsy specimens. Expression intensity values (median 25–75%) for MOR in intraoperative resected biopsy were higher in GA 8.5 (3–17) versus PPA 1 (0–10), *p* = 0.04. The numbers of MOR-positive cells were also higher in GA patients. Expression and absolute numbers of CD56, CD57, CD4 and CD68 were similar in resected tumor in both groups.

**Conclusion:**

General anesthesia with opioid analgesia increased resected tumor MOR expression compared with propofol–paravertebral anesthetic technique, but the anesthetic technique did not significantly influence the expression of immune cell markers.

## Introduction

Cancer remains the second largest cause of morbidity and mortality in the developed world. Although treatment is often focused on the primary tumor, it is the metastasis that usually causes mortality. The perioperative period is of particular importance due to a number of factors that may influence whether circulating primary tumor cells survive to develop into subsequent metastases [1].

Over a decade ago, the hypothesis was put forward that aspects of anesthetic technique might influence the outcome from cancer surgery [2]. Most clinical data published to date have been retrospective and suffer from significant confounding variables and biases. Over recent years, the need for prospective randomized controlled trials has been acknowledged.

Concurrent translational investigation has revealed that increased expression of µ-opioid receptors (MOR) in non-small cell lung cancer [3], prostate cancer [4], gastric cancer [5] and melanoma [6, 7] is associated with greater degree of metastasis [8]. Further laboratory work reveals multiple interactions between opioids and transcription factors for tumor growth, immunomodulation and vascular endothelial growth factor (VEGF) expression, leading to modulation of neovascularization [9]. This raises the issue whether opioids given during primary cancer surgery might inadvertently promote metastasatic processes.

In tandem, immunocyte concentration in peritumoral stromal tissue has been linked with the immune response to tumor cells and hence may have prognostic implications in breast [10], lung [11], gastric [12] and colorectal cancers [13]. In a meta-analysis of patients with lung cancer, high levels of CD8, CD4 and CD3 T cell infiltration into the tumor stroma showed better overall survival, whereas high density of FOXP3+ T cell infiltration was a negative prognostic factor.

While a previous report from a recent prospective, randomized pilot study [14] suggests that anesthetic technique may have a role in attenuating tumor immunocyte expression, this work was limited by its failure to compare cancer tissue removed at the time of surgery with pre-anesthetic tumor biopsy tissue. The effect of anesthetic technique on MOR expression in cancer tissue has not been evaluated in any previous prospective randomized clinical study.

Therefore, we tested the hypothesis that anesthetic technique influences tumor µ-opioid expression and the peritumoral immunocyte populations using a prospective, randomized clinical trial.

## Methods

The ongoing international multicenter clinical trial (NCT 00418457) provided the platform for this research. This trial was set up to examine if anesthetic technique had an effect on the 5-year survival in breast cancer patients. Within the framework of the study, patients were randomized into one of two groups. There were no appreciable differences in patient demographics or breast cancer morphology between the groups, including the grade and clinical stage of the breast cancers diagnosed or patients’ physiological attributes. The first group was induced with fentanyl 100 μg and propofol 200 mg (range 145–230 mg), followed by maintenance with sevoflurane at an average minimum alveolar concentration of 1.2. Intraoperative analgesia was provided by morphine with a dose range of 4–16 mg and an average of 8 mg. Postoperative pain was controlled with morphine patient controlled analgesia (PCA). The second group had a paravertebral block performed followed by a propofol-only anesthetic induction and maintenance with propofol-only total intravenous anesthesia. A paravertebral catheter was inserted using a landmark and loss of resistance technique at the level of the fourth thoracic vertebrae. Intraoperative analgesia was afforded by the instillation of 20 ml of 0.5% bupivacaine into the paravertebral space and this was followed by a paravertebral infusion of 0.125% bupivacaine in the postoperative period.

Research Ethics Committee approval was obtained to re-contact women already enrolled in the clinical trial (NCT 00418457) requesting their consent to analyze their breast cancer tissue excised during primary breast cancer surgery. Initial biopsy and surgical specimens of 20 women with biopsy-proven breast cancer, who had been previously randomized to one of two different anesthetic techniques in the ongoing clinical trial, were randomly selected from the 300 patients already enrolled in the clinical trial in our center at that time. Because this is a pilot study, 20 patients’ datasets were chosen as being likely to indicate whether any significant difference would be measurable. A table of random numbers was used, with the final two digits being used to indicate the patient number enrolled from our database. For example, if the final two digits from the random numbers table was ....71, then patient number 71 from our database of patients enrolled in the clinical trial was selected for inclusion in the present study. Twenty patients were enrolled for the present study in this way, 10 from the standard general anesthetic (GA) group and 10 from the propofol-paravertebral group (PPA). These 20 previously enrolled patients were contacted by letter containing a patient information leaflet with follow-up telephone contact by the research nurse to confirm and ascertain their consent.

All 20 patients consented to the study. Their preoperative and surgical breast cancer tissue samples were reviewed and re-stained for differential expression of markers of immunocyte infiltration and µ-opioid receptor expression. Immunocyte infiltration MOR expression of the breast cancer tissue samples was measured using immuno-histochemical analysis of tumor samples using CD4 and CD 8 markers of T lymphocytes CD 56 NK cells, CD 68 macrophages and MOR.

### Immunocyte and MOR Immunohistochemical staining

Formalin-fixed paraffin-embedded tissues (breast biopsy and post-surgery breast cancer tissue) were processed and stained for differential expression of markers of immunocyte infiltration and µ-opioid receptor expression with immuno-histochemical analysis, using CD4 (IR649, Agilent Dako) and CD8 (IR623, Agilent Dako) markers of T lymphocytes, CD56 (IR628, Agilent Dako) and CD57 (IR647, Agilent Dako) for NK cells, CD68 (IR613, Agilent Dako) for macrophages and MOR (ab137277, Abcam) as a marker for µ-opioid receptors. The immunocyte markers were ready to use antibodies (as per manufacturer) and the optimal dilution of the MOR antibody was determined by the dilution curve (1:500). Antigen retrieval (Agilent Dako PT LinK) was performed for 20 min at 97 °C in target retrieval buffer at pH 9 (immunocyte markers) and pH 6 (MOR). The slides were then stained with the respective antibody on the automated DAKO Link 48 Autostainer. Primary antibodies were incubated for 20 min (immunocyte markers) and 30 min (MOR) followed by mouse linker in the case of CD4, CD56, and CD57 and rabbit linker for MOR (Agilent Dako Envision Flex Linker kit). The chromogen, DAB (3, 3′-diaminobenzidine), was used for revelation (2 × 5 min) and counterstained with hematoxylin. Negative controls were included. The degree of staining indicated the level of infiltration.

### Data analysis

Both preoperative and postoperative samples were analyzed by two independent investigators. Cell count and staining intensity were measured. Cell count was obtained by counting the absolute number of cells visible on a ×40 magnification field. Ten distinct views at ×40 magnification were obtained and the cells counted. The median interquartile range (IQR) of these ten randomly selected high magnification fields is reported. The staining intensity is the intensity of dye retention on a 4-point scale with 0 indicating no staining and 3 representing intense staining. The staining index was calculated as the product of the mean cell count and the mean staining intensity. These data were not normally distributed and so comparison between median (interquartile range) for the two anesthetic techniques was undertaken using Mann–Whitney test.

## Results

As shown in Table 1, natural killer (NK) cells (CD56/CD57), T lymphocytes (CD4/CD8) and macrophages were present in essentially equally low levels in both groups preoperatively. There were no differences in the expression of µ-opioid receptor, CD56, CD57, CD4, CD8 or CD68 in the biopsy samples obtained before surgery between the two groups. Likewise, tumor MOR was expressed equally in both groups.

**Table 1:** Breast cancer tissue immune cell immunohistochemical expression (staining index or raw cell numbers) for preoperative biopsy and resected biopsy after anesthesia and surgical excision

Marker	Preoperative biopsy GA	Resected biopsy GA	Preoperative biopsy PPA	Resected biopsy PPA	*P* value
MOR staining index	1 (0–5)	8.5 (3–17)	0 (0–3)	1 (0–10)	0.04
MOR number of cells	5 (0–12)	14 (5–26)	6 (0–16)	7 (0–18)	0.04
CD56 number of cells	1 (0–1)	6 (0–15)	1 (0–1)	3 (0–8)	0.4
CD57 number of cells	1 (0–2)	2 (0–2)	0 (0–1)	1 (1–2)	0.8
CD4 number of cells	0 (0–1)	8 (3–20)	0 (0–1)	10 (5–23)	0.7
CD8 number of cells	0 (0–0)	3 (1–10)	0 (0–1)	3 (0–10)	0.8
CD68 number of cells	5 (1–10)	3 (0–5)	4 (1–10)	3 (0–5)	0.8

*P* values represent comparisons of the resected biopsy scores between patients who received sevoflurane–opioid anesthesia (GA group) and propofol–paravertebral anesthesia (PPA). Data shown are median (Interquartile range)

*MOR* µ-opioid receptor

Data are presented as breast cancer tissue immune cell immunohistochemical expression (staining index or raw cell numbers) for preoperative biopsy and resected biopsy after anesthesia and surgical excision. *P* values represent comparisons of the resected biopsy scores between patients who received sevoflurane–opioid anesthesia (GA group) and propofol–paravertebral anesthesia (PPA). However, resected tumor expression of MOR and absolute numbers of MOR-positive cells were increased among patients who received GA compared with PPA (Table 1). As expected by the inflammatory nature of surgery, the levels of T lymphocytes (CD4/CD8) and macrophages (CD68) were higher in the intraoperative samples than in the preoperative biopsy, but resected tumor values did not differ significantly between the groups.

In contrast, MOR levels were unaffected by surgery in the PPA group, but were significantly increased in the GA intraoperative samples (p = 0.04) when compared to the preoperative biopsy samples.

## Discussion

This is the first prospective study looking at the effect of anesthetic technique on the expression of MOR. It demonstrates that the anesthetic technique may have an effect on tumor expression of MOR, but our sample size was not large enough to detect any difference in immunocyte population between the two groups. Although our previous work in this area demonstrated an increase in immunocyte level in the GA group [15], we did not compare the intraoperative to the preoperative samples and used a color deconvolution method of analysis that has since been shown to be less accurate than the method employed in this study. There have been several reports suggesting that opioids dramatically reduce NK cell activity [16–18].

The observed difference may be due either to the effect of opioids on the tumor cells or by the reduction in stress response by the paravertebral block or indeed by the direct effect of the local anesthetic on sodium channels expressed in tumor cells, leading to a change in cell signaling. µ-Opioid receptors, the product of the OPRM1 gene, have been demonstrated on several cancer cell lines including breast cancer, non-small cell lung cancer, adenocarcinoma and gastric carcinoma [19–21]. Retrospective studies on both non-small cell lung cancer and prostate cancer have indicated that MOR expression correlates with the aggressiveness of the tumor as well as progression-free survival and overall survival [3, 18]. The mechanism for this apparent altered behavior of cancer cells depending on the density of MOR expression may be multifactorial and involve cross activation of genes which are pro-metastatic including the proto-oncogene (Src), signal transducer and activator of transcription 3, neuroepithelial cell-transforming gene 1 protein [22] and the serine/threonine-specific protein kinase (Akt) [16], all of which are expressed in tumors with high metastatic potential. It is possible that difference in immune cell expression which we observed may be caused by the interaction between the morphine and the OPRM1 gene causing an increase in MOR expression.

The nature of the immune response to cancer, surgery and anesthesia appears to be regulated by the balance of the two subsets of CD4 T helper cells, TH1 and TH2. TH1 cells secrete IFN-γ, IL-2 and TNF-α and thereby activate the ‘cellular immunity’ pathway which is responsible for protection against viruses, cancer and intracellular pathogens. In contrast to the TH1 response, the TH2 response involves the secretion of IL-4, IL-5 IL-10 and IL-13, which induce ‘humoral immunity’ thereby protecting against extracellular organisms. Optimally, the TH1/TH2 balance would be TH1 predominant in the case of cancer. TH1 release of IL-2 and IFN-γ has been shown to have strong anticancer properties by increasing the activity of CD56 NK cells. In comparison, TH2 cells have been shown to increase the levels of matrix metalloproteinase which increases the rates of metastasis by encouraging tumour cells to break through the surrounding cellular matrix. NK cells are cytotoxic lymphocytes that express a CD56 marker and recognize tumor cells without sensitization and can mount an immune response [23]. NK cells can modify the growth and dissemination pattern of certain tumor cells [24]. Research indicates that there are two subsets of NK cell dependent on the intensity of CD56 expression and the presence of the CD 16 antigen, and it is the population that stains most densely for CD56 and CD 16 that are reactive to tumor cells [25]. Our results demonstrate no discernible difference between the groups with respect to peritumoral immunocyte counts; however, it is unclear if immunocyte population size is a good surrogate for immunocyte activity.

This study has demonstrated a difference in the tumoral MOR expression. Unfortunately, we were unable to discern the mechanism for this difference. This difference in tumoral MOR expression would appear to be of prognostic significance in many cancer types. From a clinical standpoint, if further studies demonstrate a similar link between MOR expression and anesthetic technique, then it would be prudent to standardize oncoanesthesia to include the use of regional anesthetic techniques and total intravenous anesthesia.
